# A Unique Marine-Derived Collagen: Its Characterization towards Biocompatibility Applications for Tissue Regeneration

**DOI:** 10.3390/md19080419

**Published:** 2021-07-26

**Authors:** Dafna Benayahu, Yehuda Benayahu

**Affiliations:** 1 Department of Cell and Developmental Biology, Sackler Faculty of Medicine, Tel Aviv University, Tel Aviv 6997801, Israel; 2 School of Zoology, George S. Wise Faculty of Life Science, Tel Aviv University, Tel Aviv 6997801, Israel; yehudab@tauex.tau.ac.il

**Keywords:** soft corals, Sarcophyton, collagen fibers, extracellular matrix, biocomposite

## Abstract

Biomedical engineering combines engineering and materials methods to restore, maintain, improve, or replace different types of biological tissues. In tissue engineering, following major injury, a scaffold is designed to support the local growth of cells, enabling the development of new viable tissue. To provide the conditions for the mechanical and structural properties needed for the restored tissue and its appropriate functioning, the scaffold requires specific biochemical properties in order to ensure a correct healing process. The scaffold creates a support system and requires a suitable material that will transduce the appropriate signals for the regenerative process to take place. A scaffold composed of material that mimics natural tissue, rather than a synthetic material, will achieve better results. Here, we provide an overview of natural components of marine-derived origin, the collagen fibers characterization schematic is summarized in the graphical abstract. The use of collagen fibers for biomedical applications and their performances in cell support are demonstrated in an in vitro system and in tissue regeneration in vivo.

## 1. Extracellular Matrix (ECM)

Matrix proteins located in the extracellular space (ECM) form a three-dimensional network composed of polymeric macromolecules. The major macromolecules are collagen and glycoproteins, which deliver biomechanical signals to the constituting cells and provide a structural framework to the tissue. Among the different multicellular cell lineages, the ECM composition varies from providing signals for functioning in cell adhesion to regulating their differentiation. The ECM forms the tissue-niche for cell binding and regulates the stored growth factors that are released during the tissue remodeling processes. The ECM also functions as a filler in the form of polysaccharide gel or fibrous proteins within the interstitial spaces, with an additional role as a compression matrix against various stress forces. The ECM proteins consist of collagen, reticular fibers, and ground substance, of which collagen is the most common component in all animals. From lower invertebrates to mammals, collagen can appear in either a fibrillar or a non-fibrillar form. The fibrillar collagens are involved in tissue stabilization and have been identified in a variety of animal groups ranging from sponges to humans [1,2,3], while the non-fibrillar collagens are found in all tissues and are fillers in the extracellular space.

## 2. Collagens Derived from Marine Sources

In the different cnidarian classes, collagen provides a scaffold for cellular organization and plays a role in skeleton formation and bio-mineralization processes [4,5], in nematocyst function [6] and in gametogenesis [7,8]. In Hydra, collagen features as a thin ECM in the form of a basal membrane, providing support for the cell layers [9]. Mesoglea ECM is a main component in jellyfish, located between their two cell layers and forming a collagenous 3D network [10]. Anthozoans, including among others sea anemones and corals, feature a mesoglea layer of varying thicknesses, embedded with collagen fibers that reinforce the ECM filler [11]. Among cnidarians, ECM clearly presents a variety of functions through the provision of support to tissues, as well as in structuring the acellular mesoglea located between the two epithelial cell layers [12,13,14]. In soft corals, the structure of a-sulcal six mesenteries contain collagen fibers, as demonstrated for *Sarcophyton*. These collagen fibers are distinctively visible in the mesogleal pockets, thus providing structural reinforcement and stiffness and maintaining the hydrostatic skeleton of the soft coral [15,16]. Among soft corals, the epidermis is separated from the gastrodermis by the mesoglea, which contains scattered collagen fibers as well as the skeletal calcite-composed sclerites [17]. Studies on soft corals and their structural features describe unique fibers, which have been examined for potential use as a scaffolding material for biomedical application.

## 3. Identification of the Soft Coral Collagen Fibers

**General visualization**—Collagen fibers isolated from *Sarcophyton* are unique in their packing as a coiled structure inside the coral tissue and which, when stretched, present a spring-like structure [15,16,18,19]. They can be mechanically extracted from the colonies and retain their natural physical properties, in contrast to collagen harvested from mammalian sources, which loses its natural structure following the procedures after their extraction from the tissues. In coral, identified long collagen fibers were identified which feature a cord-like structure with a defined coiled morphology of various pitch sizes that provides them with the ability to stretch and retract [18].

**Figure 1:**
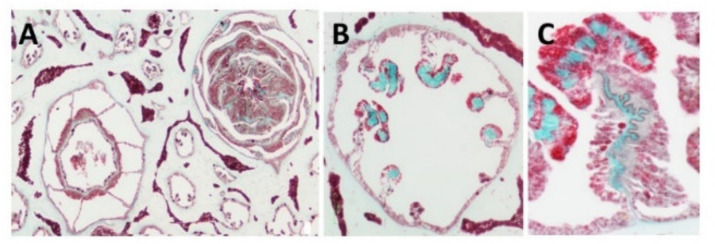
Histology cross of coral pharyngeal cavity of the polyp (**A**), six out of the eight radial mesenteries contain the collagen fibers (green, **B**) and a larger magnification of one mesentary (**C**).

**Light and fluorescence microscopy**—Histological sections of the polyp-bearing part of *Sarcophyton* positively stained with Masson Tri-Chrome revealed a green color, indicating the presence of collagen. The mesenteries below the pharyngeal cavity of the polyp in six out of the eight radial mesenteries are thicker due to the presence of the collagen fibers and are visualized in a histology cross section Figure 1 and detailed in study [15,19]. These fibers, when extracted and visualized under fluorescence microscopy, exhibit an intrinsic auto-fluorescence, detected at a range of 305–450 nm, as is typical for collagen [18].

**Figure 2:**
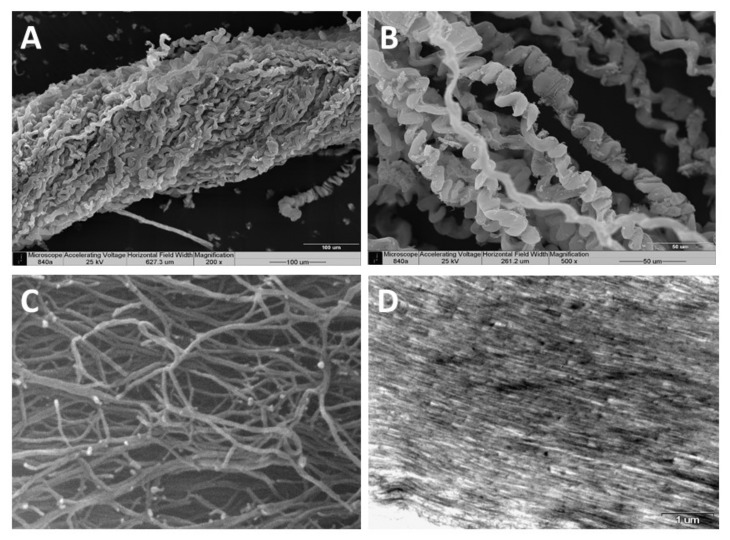
Scanning electron microscopy of the collagen fibers’ 3D organization (**A**,**B**). A pulled-out fiber’s coiled spring-like structure with natural micro crimping (**B**). Environmental scanning electron microscopy (ESEM) shows the interwoven organization of the fibrils comprising the coiled fibers and their free ends, which are either bifurcated or fused (**C**). Transmission electron microscopy (TEM) of sectioned fibers reveals their fine coiled structure (**D**).

**Scanning electron microscopy (SEM)**—SEM revealed the organized bundles of the collagen fibers, as well as the 3D features of the fibers (Figure 2A,B). When the fibers are pulled out, they reach up to several cm long, displaying a coiled spring-like structure with a natural micro crimping and a pitch range of 6–40 µm (Figure 2B). The diameter of an individual fiber is between 9–25 µm [15,19] and that of a bundle is up to several hundred microns. Interestingly, the diameter of an individual coral-derived fiber is much smaller compared to a fiber isolated from a rat-tail tendon, which measures in the range of 100–300 µm [20]. These soft-coral collagen fibrils’ micro crimping demonstrate unique structures [19,21].

**Environmental scanning electron microscopy (ESEM)**—ESEM revealed the interwoven organization of the fibrils comprising the coiled fibers and their free ends, which either are bifurcated or fused (Figure 2C). The ESEM images also revealed a fibril diameter of 25 nm [19], being smaller than the known diameter of rat-tail fibrils (10–500 nm) [22].

The defined coiled spring-like structure with natural micro crimping and either bifurcated or fused structural features underlie the hyper-elastic mechanical properties that enable the unique response of soft-coral collagen fibers as detailed below.

**Transmission electron microscopy (TEM)**—TEM of sectioned fibers (Figure 2D) revealed their fine coiled structure, and a parallel arrangement of fibrils with a repeated pattern of dark and light bands perpendicular to the fibril axis, similar to the well-known fibrillary collagen striation [19,21]. The mesoglea of *Sarcophyton* also contains short and sparse collagen fibrils located between the long fibers, as is commonly found among other cnidarians [19,21].

**X-ray fiber diffraction**—X-ray diffraction confirmed the structural arrangement of the soft-coral collagen fibers, showing them to possess a meridional periodicity of 66 nm and a triple helical structure, as studied in both forms of wet or dried fibers, similar to the fibers of rat-tail tendons. The X-ray analysis revealed the phases of mixed type I and type II collagen and validated the TEM results of the D-period of the collagen fibers’ structure [21]. The electron-dense profile visualized on the soft-coral fibers, as well as for vertebrate isolated fibers, is related to type I and II collagens, indicating both a strong resemblance between them, and also to helical organization of Type I. These features may derive from the unique amino acid composition or the deposition of other proteins, such as proteoglycans, on the surface of the collagen fibrils and the fibril-bundles [21].

**Molecular analysis**—The microstructural features of *Sarcophyton* fibers revealed by histology, TEM, and X-ray analysis confirmed the collagenous features of the coral fibers. Additionally, molecular confirmation was acquired by tandem mass spectrometry (MS/MS) using MALDI-TOF-TOF to determine the nature of the protein. The peptide characterization of the fibers’ organic matrices, based on amino acid sequencing, confirmed them to be collagen type I-II. The results of the Uniprot comparison indicates that the identified peptides are homologous to those of other organisms, and thus confirmed their evolutionarily conserved protein structure. Among vertebrates, 29 collagen types are recognized; for cnidarians, however, the available data are limited (https://www.ncbi.nlm.nih.gov/protein/, accessed on 25 July 2021). In invertebrates, most of the organisms subjected to molecular analysis to date have been sea anemones and jellyfish, while for others only partial sequences have been reported (in the RefSeq Protein Database, NCBI). Moreover, the majority of collagen and collagen-like proteins described for invertebrates belong to the non-fibrillary and soluble structure subtype IV, and only a few identified collagens are associated with type I. A comparison of the peptide sequences obtained for the *Sarcophyton* collagen by the MS/MS assay with that of the UniProt database reveals a remarkable homology of the soft-coral collagen to the fibrillary mammalian one [19,21]. The MS/MS results correspond to the results of the proton and carbon NMR amino acid analysis, which confirmed the presence of a high concentration of glycine and hydroxyl-proline in a ratio corresponding to that of a collagenous protein [15,16,21].

**Differential scanning calorimetry (DSC)**—Differential scanning calorimetry (DSC) conducted on the *Sarcophyton* fibers indicated that the collagen exhibits an unexpectedly high denaturation temperature of ~67.8 °C. In contrast, native type I and II collagens exhibit a value of 42 °C for soluble tropo-collagen, which increases to 54 °C upon fibrillation, or to 67 °C when it is glycamix cross-linked. The analysis of the soft-coral fibers determined their melting point to be 68 °C [15,16], indicating that their natural cross-linking also contributes to their exceptional mechanical properties as described below, relying on a series of previous studies [22,23,24,25]. It seems, therefore, that the soft-coral fibers indeed constitute a unique mesoglea component, featuring a structural similarity to vertebrate tendons or ligaments rather than to those of other cnidarians.

## 4. The Application of Marine Collagen Fibers as Scaffolding for Tissue Engineering

Tissues comprise an assembly of biomaterials that form a heterogeneous ECM suited to each type of tissue. Tissue composition is highly adapted to both biological and mechanical functions. Tissue engineering, therefore, focuses on developing a scaffold for repair that will support tissue regeneration. Consequently, the scaffold should resemble as closely as possible the properties of the native tissue. The tailor-made mechanical properties of a tissue should be carefully considered in order to meet the required tensile or compression loading forces. Collagen is the major protein that functions as a load-bearing element in tissues, in addition to its function in cell adhesion. Collagen-based biomaterials are therefore fundamental for tissue engineering purposes.

Mammalian-derived collagen has been widely used to produce scaffolds, but this requires tedious processing along with cell-removal procedures. In most cases, the fibrous macro-structure of the resultant collagen is damaged, or even destroyed, thus leading to reduced strength compared to the natural collagen. Over the years, diverse techniques for strengthening these collagen fibers and/or improving their formation have been designed in order to reproduce the complexity of the native collagen, including an intensive exploration of collagen derived from marine sources [15,16,18,19]. Efforts to produce human-derived collagen and overcome the immunological response using the biotechnology approach and in genetic engineering have used the expression of human genes in tobacco plants for the mass production of collagen; However, this is highly expensive to produce [26]. The marine-derived collagen has the advantages of a reduced immunogenic response, lower cost, and a lack of associated ethical issues involved in its application. Thus, the use of coral collagen as a biomaterial, along with other constituents to form an ECM, plays a promising role toward scaffolding production for tissue repair and regeneration. The stiffness of the coral fibers conduces to mechanical robustness [23,24,25,27] and oriented cell growth [18], while the addition of hydrogels to the biomaterial provides a soft and aqueous environment that benefits the resident cells in the recovering tissue and aids the formation of a 3D structure similar to that of the natural tissue [28,29].

## 5. A Tailored Bio-Composite Composed of Natural Marine Components

**Figure 3:**
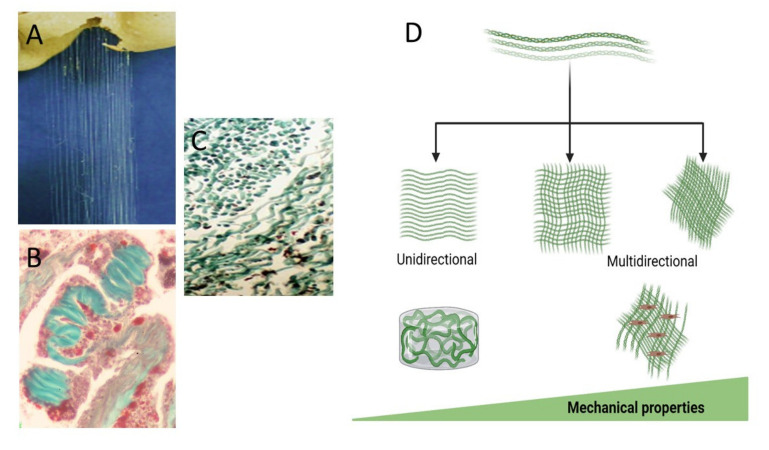
Figure **3.** The collagen fibers extracted from the soft coral (**A**), masson trichrome stained fibers in the mesentery (**B**), and the isolated fibers (**C**). The fibers are used for the arrangement in various orientations can be unidirectional or multidirectional and tailor-made for the desired biomechanical properties designed according to tissue needs for applied forces and a different 3D bio-composite (**D**).

The bio-composite material, composed of an all-natural marine-derived source, is constructed from the *Sarcophyton* collagen fibers embedded in an alginate hydrogel matrix [18,28,29]. The use of alginate is common for tissue engineering purposes, as well as for encapsulation and drug delivery. The ability to exploit the collagen fibers’ particular orientation when embedded in hydrogel, either in a unidirectional or multidirectional manner, is schematically illustrated in Figure 3. Such a bio-composite is tailor-made to the desired biomechanical properties and achieved by aligning the fibers on a frame immersed in an alginate and a physiological solution containing calcium for the cross-linking gelation. This multi-step procedure of fibers embedded in an alginate gelation and cross-linked via ionic bridges binds the two components into a bio-composite.

The benefit of using the soft-coral collagen fibers derives from their simple isolation, macro-fibrous structure, ultra-long length, and superior mechanical properties as a biopolymer [23,24,25,27]. The design and tailoring of a bio-composite is based on the orientation of the fibers, thus enabling the pre-determined mechanical-properties. The tensile measurements that were analyzed under digital image correlation (DIC) in a series of experiments, allowing us to test the structural integrity of the tailored bio-composite. Studies of the mechanical properties revealed a uniform pattern that was maintained when analyzed in a series of cycles with stretching forces up to 10% strain. The obtained mechanical properties were compatible with those known for load-bearing functional tissues, such as those of the aorta and cornea [27].

## 6. Bio-Composite Production and Its Biocompatibility

The biocompatibility of the coral collagen-based scaffold was demonstrated to support cell growth both in vitro [18,28] and in vivo [29]. The algenic acid in the presence of calcium ions produced a hydrogel that also contributed to the 3D structure and ECM-like material. The collagen fibrils enable cell functionality based on interacting structural functions facilitating cell-interacting domains that sustain cell adhesion and growth towards tissue remodeling, which yields a successful scaffold and the development of new tissue. The regulation of cell adhesion to a given scaffold is related to binding via integrin receptors in the presence of von Willebrand factor type A (vWFA), a metal ion-dependent adhesion site, and the fibronectin type 3 domain [30]. Additionally, the immunoglobulin-like component, cytokine receptor motif, and integrin comprise the main family of cell adhesion molecules, functioning to transduce signaling in and out of the cell by linking the cytoskeleton to the ECM proteins. The laminin G domain motif also plays a role in the cell adhesion, signaling, migration, and differentiation [31]. Thus, the soft-coral fibrillar collagen possesses properties that strongly support cell adhesion, making it a highly suitable component for a successful scaffold. The collagen fibers allow cell attachment and spread, and thereby support their growth [17,26]. In addition, the collagen fibers and their elastomeric features are capable of enduring significant physical deformation without breaking, and of retracting to their original conformation onc the physical stress is removed [32]. The outstanding mechanical properties of the described soft-coral collagen fibers are attributed to their elastomeric nature [23,24,25,27]. The proteins’ nature, along with the mechanical properties of the *Sarcophyton* fibers, makes them a suitable biomaterial for scaffold applications, in contrast to the synthetic electro-spun fibers, such as poly-lactic-co-glycolic acid (PLGA) [33]. Because PLGA does not possess cell attachment properties, its biomimetic ability is therefore lower than that of the soft-coral collagen [18,28]. Other collagen sources, such as jellyfish collagen, are not fibrillar and thus lack the biomechanical properties of the *Sarcophyton* fibers [33,34]. The importance of a hybrid scaffold formation possessing biomimetic properties, in particular cell biocompatibility and tissue strength, is of major significance for the fibers derived from the soft coral. Although the marine source creates the bio-composite components, being evolutionarily remote from vertebrates, the collagen is a well-conserved protein [1]. Thus, the usage of the coral collagen fibers opens a new opportunity, and the unusual biomechanical properties of these fibers allow us to develop a hybrid composite that can be determined by applying different densities of the collagen fibers to the bio-composite (Figure 3). The stiffness of the scaffold depends on the collagen orientation and the concentration of the alginate. No less important are the supporting cues necessary for cell activation, growth, and fate, as demonstrated in an in vitro study on mesenchymal stem cells [18,28]. Clearly, the soft-coral-derived scaffold enabled a successful cell migration and colonization of the fibers, leading to cell proliferation and tissue-like structures along and between the collagen fibers [18,28,35]. The collagen–alginate scaffold demonstrated a superior mechanical compatibility (e.g., strength and elasticity) [22,23,24,25] alongside its biocompatibility properties [18,28,29]. The biocompatibility of the collagen–alginate, which was extensively assessed in vitro, demonstrates that the scaffold meets the terms of the IS0 10993-5 standards for cytotoxicity [18,28].

## 7. A Bio-Composite for In Vivo Tissue Regeneration

The tailored scaffold from collagen fibers in alginate hydrogel offers great potential for application in a variety of tissue grafts [29]. This bio-composite material mimics the components of the natural tissue based on the collagen fibers that provides the load-bearing needed while the hydrogel provides the ECM-like properties for the cells’ environment. The effective mechanical impact of the bio-composite lies between the fibers and the matrix, in which the local mechanical properties are stiff enough to support new tissue formation in the hybrid combining the two constituent materials, inspired by the structure of natural tissues. Tailoring the mechanical behavior of the bio-composite to the targeted tissue can be achieved by altering the collagen fiber fraction and orientation, which have been shown to yield different mechanical properties [23,24,25,27,33,35,36]. Following a study on biocompatibility for cell growth and their differentiation, the in vitro results indicated a lack of cytotoxicity for a period of up to several weeks [18,28,29]. The collagen–alginate bio-composite, when transplanted into rats [29] was evaluated while establishing contact with several tissues. At the subcutaneous site, it was adjacent to the skin hypodermis, and when next to a tendon and a muscle it was adjacent to a rich vascularized tissue. An extracted scaffold from the transplanted sites in rats revealed a stable bio-composite film and the subsequent healing process was successful. Throughout the period of the transplanted bio-composite, the rats were monitored for their general health, appetite, and an increase in weight. In addition, their normal activity and non-aggressive behavior indicated a lack of stress or suffering, up to a complete cure with no signs of cytotoxicity. All the operated rats displayed improved mobility during the recovery period, with no difference between operated or non-operated limbs. The results of the in vivo experiment thus revealed that the bio-composite could serve as an augmenting scaffold while also protecting the tendon by allowing tissue regeneration and transplant biocompatibility. The transplants integrated with the surrounding tissues and a capsule of granulation tissue formed, indicating enhanced fibrogenesis as part of the healing process and new tissue formation. The conclusion from the in vivo experiment was that the soft-coral collagen-based bio-composite had no negative effect on an animal’s health, with the results of the assay corresponding to the in vitro cytotoxicity results [28]. The bio-composite and its safety is a principal quality for future clinical applications and meets the ISO standard for biocompatibility in vitro and in vivo.

## 8. Summary

The importance of the formation of scaffolding for biomedical applications lies in its ability to mimic the tissues’ mechanical properties. This ability to mimic natural tissue is essential in all scaffolding intended to provide the biological properties needed for cell attachment, as well as to enable transduction of the signals that trigger cells to attach and spread across the collagen fibers. Such interactions between the cells and the scaffold activate the cytoskeleton in response to the cells’ tensional forces, leading to tissue-like formation [18,28,29,37]. When using mammalian collagen, the need for chemical processes of extraction, isolation, purification, and polymerization together reduces its natural properties. Additionally, collagen derived from a bovine or porcine source also introduces a risk of pathogens. The marine sources of collagen are mainly from jellyfish, and while such collagen has proven its biocompatibility and supports cell viability [38,39], it possesses poor mechanical properties and is thus of limited applicative use [40,41].

The unique collagen fibers harvested from the soft coral overcome some of these issues as described for the novel approach using the scaffold biomaterial composed of collagen fibers and alginate hydrogel, both from marine sources. Both these marine-derived materials support cell growth, differentiation (in vitro), and tissue transplants (in vivo) and exhibit no toxicity [18,28,29]. The outcome of the in vivo transplanted biomaterial demonstrated the biocompatibility, a good integration of the bio-composite, and led to a good recovery of the animals. The transplanted scaffold enabled the formation of a 3D structure and also facilitated cell migration and new blood vessel formation, both of which are required for tissue repair.

The novel bio-composite enables the tailoring of specific mechanical properties alongside the biological properties required in order to mimic the structure of natural tissue. Many of the known soft tissues in humans and other animals demonstrate a variable density in their collagen-fiber component, according to their specific function, such as for cartilage and abdominal wall repair, blood vessels, and cardiac tissue, thus yielding what is necessary for a large variety of tissue engineering applications.

We present here Table 1 which summarizes the main properties of collagen from the coral source.

## Figures and Tables

**Table 1:** Summary of the methodologies applied to examine the properties of the *Sarcophyton* collagen fibers.

Methodology Used	
*Morphology*	Text Reference
Light microscopy confirming location and collagenous nature	[15,19]
Fluorescent microscopy	[18]
Scanning Electron Microscopy (SEM) demonstrating fiber coiling	[15,19]
Environmental scanning electron microscopy (E–SEM) demonstrating fibrilar structure	[19]
Transmission electron microscopy (TEM) demonstrating fibrilar micro structure	[19,21]
***Physical properties***	
Fibers diameter 9–25 µm	[15,19]
Fibrils diameter 25 nm	[19]
DSC 68 °C	[15,16]
***Molecular properties***	
Histology staining (Masson Trichrome)	[15,19]
Nuclear magnetic resonance (NMR) revealing high levels of Glycine and Hydroxy-proline)	[15,16,21]
Maldi–TOF (MS/MS) sequence analysis	[21]
X-ray diffraction revealing (periodicity of 66 nm	[21]
***Cytotoxicity and biocompability***	
In vitro cell growth	[18,28]
In vivo transplanted scaffold	[29]
